# Improving nursing documentation for surgical patients in a referral hospital in Freetown, Sierra Leone: protocol for assessing feasibility of a pilot multifaceted quality improvement hybrid type project

**DOI:** 10.1186/s40814-021-00768-5

**Published:** 2021-01-27

**Authors:** Nataliya Brima, Nick Sevdalis, K. Daoh, B. Deen, T. B. Kamara, Haja Wurie, Justine Davies, Andrew J. M. Leather

**Affiliations:** 1 King’s Centre for Global Health & Health Partnerships, School of Population Health & Environmental Sciences, Faculty of Life Sciences and Medicine, King’s College London, London, UK; 2 Centre for Implementation Science, Health Service and Population Research Department, Institute of Psychiatry, Psychology and Neuroscience, King’s College London, London, UK; 3 Connaught Teaching Hospital Complex, Freetown, Sierra Leone; 4 Department of Surgery, College of Medicine and Allied Health Sciences, University of Sierra Leone, Freetown, Sierra Leone; 5 Faculty of Nursing, College of Medicine and Allied Health Sciences, University of Sierra Leone, Freetown, Sierra Leone; 6 Institute of Applied Health Research, University of Birmingham, UK; Centre for Global Surgery, Department of Global Health, Stellenbosch University, Stellenbosch, South Africa

**Keywords:** Surgical patient records, Nursing notes, Surgical patients, Quality improvement (QI) intervention, Implementation outcomes, PDSA, Evaluation of outcomes

## Abstract

**Abstract:**

**Background:**

There is an urgent need to improve quality of care to reduce avoidable mortality and morbidity from surgical diseases in low- and middle-income countries. Currently, there is a lack of knowledge about how evidence-based health system strengthening interventions can be implemented effectively to improve quality of care in these settings. To address this gap, we have developed a multifaceted quality improvement intervention to improve nursing documentation in a low-income country hospital setting. The aim of this pilot project is to test the intervention within the surgical department of a national referral hospital in Freetown, Sierra Leone.

**Methods:**

This project was co-developed and co-designed by in-country stakeholders and UK-based researchers, after a multiple-methodology assessment of needs (qualitative, quantitative), guided by a participatory ‘Theory of Change’ process. It has a mixed-method, quasi-experimental evaluation design underpinned by implementation and improvement science theoretical approaches. It consists of three distinct phases—(1) pre-implementation(project set up and review of hospital relevant policies and forms), (2) intervention implementation (awareness drive, training package, audit and feedback), and (3) evaluation of (a) the feasibility of delivering the intervention and capturing implementation and process outcomes, (b) the impact of implementation strategies on the adoption, integration, and uptake of the intervention using implementation outcomes, (c) the intervention’s effectiveness For improving nursing in this pilot setting.

**Discussion:**

We seek to test whether it is possible to deliver and assess a set of theory-driven interventions to improve the quality of nursing documentation using quality improvement and implementation science methods and frameworks in a single facility in Sierra Leone. The results of this study will inform the design of a large-scale effectiveness-implementation study for improving nursing documentation practices for patients throughout hospitals in Sierra Leone.

**Trial registration:**

Protocol version number 6, date: 24.12.2020, recruitment is planned to begin: January 2021, recruitment will be completed: December 2021.

**Supplementary Information:**

The online version contains supplementary material available at 10.1186/s40814-021-00768-5.

## Background

Over 5 billion people in the world do not have access to safe, affordable, and timely surgical and anaesthesia care when needed [1]. While surgery can save lives and prevent disabilities, it can also result in perioperative death and complications, with patients in low- and middle-income countries (LMICs) most at risk [1–4]. The recent ‘Lancet Global Health Commission on High Quality Health Systems in the Sustainable Development Goals Era’ report has highlighted that the quality of health care in LMICs is poor and that quality might present a bigger challenge than access in reducing mortality and disability [2].

Mortality after surgery in Africa is two times higher than the global average, despite patients being younger, having a lower-risk profile, and developing fewer complications [3]. The risk of surgical site infections (SSIs) in African surgical patients is also higher than that reported in USA and Europe (15.5% vs 2.8%/pooled per 100 surgical patients, respectively). SSI remains the highest hospital-acquired infection and the most common post-operative complication [3–5]. Improving quality of surgical care needs to be prioritised in order to reduce avoidable mortality and morbidity [2]. To provide high-quality surgical care, health systems in sub-Saharan Africa and other LMIC settings need to be strengthened [6].

High-quality surgical care requires effective communication across a multidisciplinary team [7–10]. A basic pillar of effective communication is an accurate patient record that provides a detailed account of the care a patient receives, ensures continuity of care, and demonstrates fulfilment of duty of care [11–13]. Nurses play a pivotal role in delivery of high-quality patient care [9]. Nursing documentation provides a means of communication and sharing of relevant information between health care professionals and facilitates evidence-based healthcare decisions that are key components of good clinical practice [12]. Good nursing documentation practice (complete, accurate, and timely) is a professional responsibility and plays a vital role in the delivery of effective and safe evidence-based health care and in quality improvement (QI) work [13–15]. Failure in surgical nursing documentation and communication results in adverse surgical patient outcomes and may lead to a range of consequences, from delays in treatment and provision of inadequate care, to adverse events such as wrong site surgery [7, 12, 13, 16].

Despite wide recognition of the importance of good nursing documentation practices, the quality of such documentation in LMICs remains poor. There are many facets to the problem including missing, insufficient, and unclear information in clinical records; poor completion of forms; incorrectly recorded patient identifiers with missing signatures, dates, and times; and insufficient use of discharge summaries and provision of care plans [7, 17–21].

In recent years, numerous efforts aiming to improve the quality of nursing documentation have been undertaken. Evidence-based approaches to improving documentation include interventions such as national clinical audits with data feedback [21, 22], the availability of documentation policies and guidelines [10], information sharing [23], provision of training [24–26], strengthening nursing hospital leadership, engaging key staff into improvement processes [9], and the use of a framework for recording nursing daily free-text notes [26, 27]. Nursing documentation can also be improved by indirect workforce and structural interventions, such as increased nurse to patient ratios and addressing equipment shortages, as these interventions make it easier for nurses to complete documentation [22]. Lastly, replacement of paper-based with electronic documentation has now been reported in the literature, with some evidence indicating that electronic documentation in hospital settings can save nurses time, reduce documentation errors, and result in better clinical outcomes such as reduced infections rates [28].

Of these interventions for the improvement of nursing documentation, only some are suitable for implementation in LMIC health care settings. For example, electronic data record systems are not easily implementable in many LMICs due to unreliable computer networks and equipment, low computer literacy level amongst health care staff (HCS), and high costs [28]. As a result, paper-based nursing documentation is generally used in LMICs and is a major target for improvement. To our knowledge, to date, there are no published QI studies aimed at improving the quality of nursing documentation in LMIC settings—despite the relevance of such approaches.

This pilot aims to assess the feasibility of delivering and assessing a set of evidence-based interventions to improve paper-based nursing documentation for surgical patients in the surgical wards of one hospital in Sierra Leone [29, 30]. The results will inform the design of a large-scale effectiveness-implementation hybrid II study [31] that will evaluate both effectiveness and implementation outcomes and that will be deployed and tested in hospitals throughout Sierra Leone.

## Methods

### Study design

This is the feasibility pilot [30] of a prospective mixed-methods, quasi-experimental hybrid study which assesses simultaneously effectiveness and implementation outcomes [31].

**Fig. 1:**
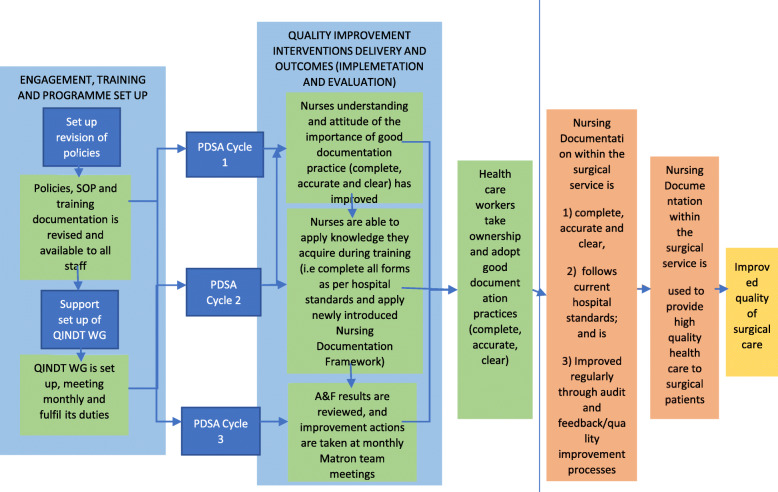
The theory of change for the nursing documentation improvement study (compact version)

The study was co-developed and co-designed during 2019 by hospital HCS and UK-based global health researchers in close collaboration with local experts in the field and Ministry of Health and Sanitation of Sierra Leone staff (MoHS). During the co-development process of this project, emphasis was put on the assessment of the needs, identification of contextual determinants to implementation, and careful selection of interventions appropriate for the local context. This process was guided by the Theory of Change (ToC) approach that can facilitate the development of complex theory-driven interventions [32]. To ensure a high level of shared understanding amongst all stakeholders involved in the process of how a service-level intervention may work in practice, a ToC map was iteratively developed and refined (Fig. 1). Full details of development and design of this study and results of the baseline assessments will be reported in detail elsewhere.

Implementation science is a research methodology that uses theoretical approaches (theories, models, and frameworks) to understand the components and implementation strategies necessary to promote context-relevant adoption of evidence-based interventions, to increase their effectiveness [33]. The implementation strategies [34] to deliver this intervention were selected using the Expert Recommendations for Implementation Change (ERIC) taxonomy of implementation strategies [35]. Out of 73 strategies listed, 52 were incorporated into this project, and most of them are ranked as both important and feasible in a concept mapping study by Waltz et al. [36].

In addition, to support the delivery of the QI process within a complex health care environment that changes over time in predictable and unpredictable ways, improvement methods can be used [37]. Plan-Do-Study-Act (PDSA) is an improvement cycle that offers a mechanism for iterative development and scientific testing of improvements in complex healthcare systems in which an hypothesis for improvement is formulated and an intervention is designed (Plan), the intervention is implemented (Do), the data is collected and results are analysed and interpreted (Study), and a plan for what to do next is determined and acted upon (Act). This approach was incorporated in the design [35]. Such methods have been shown to enable improvement of health care processes and patient outcomes, if they are applied with good fidelity [38–40].

To optimise the evaluation of the outcomes of this project, two formal evaluation frameworks will be used, the Kirkpatrick Learning Evaluation Model [41] and the implementation outcomes taxonomy proposed by Proctor et al. [42]. Kirkpatrick’s model is suitable for the evaluation of training programmes, such as the one that will be used within this study. This model refers to four levels of evaluation outcomes: reaction, learning, behaviour, and results. A training programme is deemed to be effective when the trainees are satisfied (level 1), they learn what they intended to learn (level 2), they behave more efficiently and skilfully at work (level 3), and the organisation benefits from the use of what individuals have learned (level 4).

The implementation outcomes taxonomy proposed by Proctor and colleagues [42] proposes that the success of an implementation effort can be determined by assessment of several subjective and objective criteria including the following:

- Acceptability: perception amongst stakeholders that the new intervention is agreeable.
- Adoption: intention to apply new intervention.
- Appropriateness: perceived relevance of the intervention for the setting and problem.
- Feasibility: extent to which an intervention can be applied.
- Fidelity: the proportion of management protocol components completed as intended.
- Coverage: the proportion of eligible patients who actually receive the intervention.
- Cost: costs of the intervention, including the delivery strategy.
- Sustainability: extent to which a new intervention becomes routinely available/is maintained post-introduction.

To ensure consistent defining and reporting of the study, Standard Protocol Items: CONSORT Extension to pilot and feasibility studies and Recommendations for Interventional Trial (SPIRIT) checklists adapted to protocols of pilot and feasibility studies, have been applied [43–45].

### Study duration

This pilot study will last for one year (planned start-end date: Jan-Dec 2021)

### Study setting

The study will take place within the surgical department of Connaught Hospital, the main referral hospital in Sierra Leone for both adult and paediatric surgery (excluding obstetrics).

There are 6 surgical wards that are included in this study (2 male, 2 female, 1 paediatric, and 1 trauma).

**Fig. 2:**
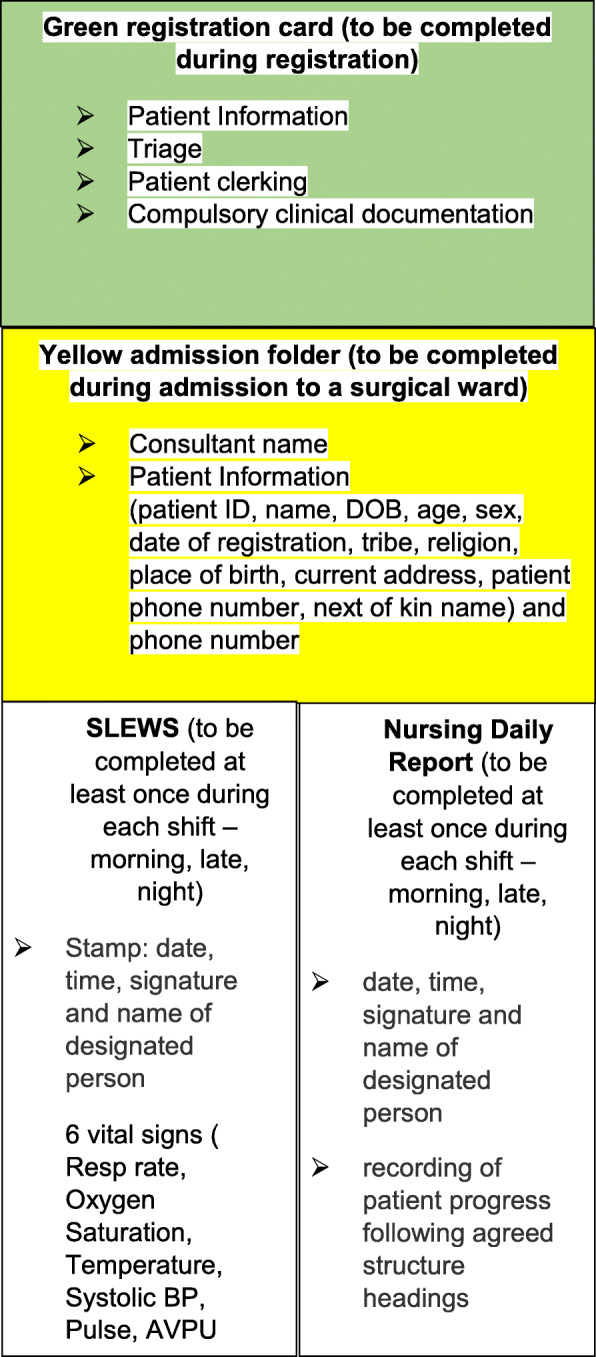
Diagram of nursing documentation within surgical patient’s folder at Connaught Hospital, Freetown, Sierra Leone

Nursing documentation forms currently being used at Connaught Hospital include the Green registration card which is completed at registration by administration staff, and in triage by triage nurses and doctors; the Yellow admission folder which is completed by ward nurses during admission onto a surgical ward; vital sign recordings on the Sierra Leone Early Warning Score (SLEWS) form; and Nurses Daily Report form (daily written notes) that are both completed by ward nurses on a daily basis (Fig. 2, Table 1).

**Table 1:** List of clinical forms and sections to be assessed in the intervention

Name of the medical form	Name of the section on the medical form	List of the fields
Hospital registration card (Green card)	Patient details section	Patient ID, name, DOB, age, sex, date of registration, time of registration, occupation, tribe, religion, place of birth, current address, patient phone number, next of kin name, and phone number
	Triage section	Date, time, and nurse name or signature
	Patient clerking and compulsory clinical documentation sections	Date, time, grade, name of doctor, contact of doctor, signature of doctor
Surgical admission folder (yellow folder)	Cover page	Consultant name, patient ID, name, DOB, age, sex, date of registration, tribe, religion, place of birth, current address, patient phone number, next of kin name and phone number
SLEWS	One entry for each shift	Date, time, name, and signature of designated person, all 6 vital signs (respiratory rate (RR), oxygen level (%), temperature, blood pressure (BP), pulse, alert-verbal-pain-unresponsive (AVPU) level, SLEWS score, and the time and name of doctor that has been called
Nurses daily report	Compulsory heading fields on each page	Patient ID, DOB/age, sex, name
	Application of Nursing Documentation Framework (a new framework to be introduced by this project), to be completed on each shift	Date, time, signature, and name of designated person; quality note recording under a set of agreed headings (to be determined)

### The project

The project consists of three distinct parts: pre-implementation, intervention implementation, and evaluation (Table 2)

**Table 2:** Phases of the quality improvement project—summary

Phase name	Phase main components	Timeline
Pre-implementation	1. Set up of QINDT 2. Equipment donation 3. Review of hospital currentnursing documentation policiesand forms	January–December 2020
Intervention	1. Awareness drive for all hospital HCS 2. Training package for surgical nurses 3. Audit and feedback process for nursingdocumentation Each is delivered using the PDSA cyclemethodology	January 2021–February 2021 March 2021–April 2021 May–June 2021
Evaluation of the entire project	Baseline data collection Endline data collection Sustainability assessment	Pre-intervention Post-intervention 6 months post-intervention

*There will be one PDSA cycle for each of the three interventional components. However, each PDSA cycle will be repeated if further improvement needs to be made

### Pre-implementation

The pre-implementation phase has been completed and included the following activities.

#### Setting up a quality improvement for nursing documentation team

The QINDT consists of a working group and nursing champions who will support the delivery of the project. The QINDT (working group and nursing champions) will be provided with training and support by the research team for the duration of this project, through regular meetings, workshops, presentations, and written reference materials. Further details on composition and remit of the QINDT are available in Appendix 1.

#### Equipment donation

One of the barriers that were identified during the intervention development process was lack of equipment on the wards to measure vital signs, such as blood pressure machines, oximeters, and thermometers. To address this structural gap, all six wards that are part of this project were provided with a set of equipment to enable the nurses to measure vital signs.

#### Reviewing and updating of the current nursing documentation policies and forms

Another structural barrier that was identified and explored during the intervention development process was the need to review and update current policies and forms related to the nursing hospital documentation practices. As a result, members of the hospital medical records department, matron’s office, and the surgical department, with support from the research team, have reviewed and updated the current nursing documentation policies and forms. One of the main changes to be introduced as part of this project will be use of a nursing documentation framework that will be recorded on nursing daily reporting forms. The updated forms will be used in the intervention implementation phase.

### Intervention implementation

The intervention consists of three components. Each is delivered by use of a PDSA cycle and is focused on a specific element: raising awareness of high-quality documentation (PDSA1); improving knowledge, skills, and attitudes around documentation (PDSA2); and finally, introducing an audit and feedback mechanism to support sustainable implementation (PDSA3). These are described in more detail below.

### PDSA1: raising awareness of the importance of high-quality clinical documentation

#### Aim

To introduce simple and cheap educational interventions that will raise awareness amongst HCS of the importance of quality clinical documentation in all groups of HCS within the surgical department.

#### Objective

Deliver, assess, and adjust the awareness drive (WhatsApp messages and educational posters).

#### Plan

The awareness drive will consist of two parts—WhatsApp texts and educational posters that will be implemented simultaneously. The content and design of WhatsApp texts and posters will be co-developed by the research team and QINDT using infographics.

#### Do

WhatsApp communications will be sent to all staff within the surgical department on a weekly basis, using existing WhatsApp groups. Educational posters will be displayed in all clinical areas (registration area, triage, SOP, outpatient, surgical wards, operating theatres, staff rooms, meeting rooms) to act as a visual aid reminder. Educational posters will be regularly checked to make sure they are in suitable places and in good condition. These activities will be undertaken by the research team.

#### Study

After 3 weeks from the beginning of the awareness drive, several outcomes to assess feasibility of delivering the intervention and capturing learning, process, and implementation will be collected and analysed as detailed in Tables 3 and 4.

**Table 3:** Feasibility outcomes

PDSA1 - awareness drive	
1) WhatsApp messages were sent to all staff and educational posters were displayed in all clinical areas, within the surgical department on a weekly basis from the start of the cycle for three consecutive weeks (yes/no)	
2) WhatsApp messages were sent to all staff and educational posters were displayed in all clinical areas, within the surgical department on a weekly basis from the start of the cycle for three consecutive weeks (yes/no)	
3) The required number of responses to assess the awareness drive has been collected for each type of outcome (implementation and process, as described in Table 4 and Fig. 3), within the time allocated (yes/no).	
PDSA2—training package:	
4) The training workshop and at least two supervision sessions have been delivered within the time allocated to at least 90% of potential participants (yes/no)	
5) The required number of responses to assess the training package has been collected for each type of outcome (implementation and process, as described in Table 4 and Fig. 3), within the time allocated (yes/no).	
PDSA3—audit and feedback:	
6) The audit and feedback cycle has been set up by the end of PDSA2 and has been undertaken by the hospital matron team at least two times within the time allocated (yes/no).	
7) The required number of responses to assess the audit and feedback cycle has been collected for each type of outcome (implementation and process described in Table 4 and Fig. 3), within the time allocated (yes/no)	
8) The research team was able to access patient files and capture the data needed to evaluate effectiveness of this project at all three time points (baseline, endline, and for sustainability at 6 months after implementation) (yes/no).

**Table 4:** Outcomes for the project by type

Outcomes	Type of outcome/description	Measurement method(s)/tools	Measurement time point(s)/time period
**Implementation outcomes**			
Acceptability	Perception amongst stakeholders that the intervention is agreeable	AIM questionnaire [46] and FGD	After each PDSA cycle
Appropriateness	Perception amongst stakeholders of the fit and relevance of the intervention to the local context	IAM questionnaire [46] and FGD	After each PDSA cycle
Feasibility	Extent to which an intervention can be successfully performed	FIM questionnaire [46] and FGD	After each PDSA cycle
Uptake of nursing documentation framework (adaptation)	% of daily nursing reports that are recorded using nursing documentation framework	Pre-existing data collection of information from patient notes/RedCap Tool	After PDSA2, PDSA3, endline, sustainability assessment 6 months after the end of the project
Fidelity of application of nursing documentation framework	% of each section completed within the framework Information recorded within each section is relevant	Pre-existing data collection of information from patient notes/RedCap Tool	After PDSA2, PDSA3, endline, sustainability assessment 6 months after the end of the project
**Implementation strategy outcomes**			
Accessibility of information	To find out if the posters were seen and WhatsApp messages received	Awareness drive questionnaire	After PDSA1
Impact of information	Perception of the messaging and posters, the impact that the messaging and posters had on you (remember, navigate, easy-to-use information)	Focus group discussion (FGD)	After PDSA1
Impact of supportive supervision	Perceptions of nurses to 1-to-1 sessions with supervisors	PSS questionnaire [47], qualitative interviews	After PDSA2
Impact of audit and Feedback cycle	Perceptions of health staff to audit and feedback	Qualitative interviews	After PDSA3
**Process outcomes related to training**			
Level 1: reaction	Extent to which participants find the educational materials and delivery of them to be favourable, engaging, and relevant to their job	Awareness drive and training workshop questionnaire	After PDSA1, PDSA2
Level 2: learning	Extent to which participants acquire the intended knowledge, skills, attitude, confidence, and commitment to good documentation practice	Awareness drive and training workshop questionnaire	After PDSA1, PDSA2
Level 3: behaviour	The extent to which participants apply on the job what they learned	Pre-existing data collection of information from patient notes/RedCap Tool	After each PDSA (data collected as part of compliance and completeness outcomes listed above)
**Effectiveness outcomes**			
Compliance of Nursing daily report	% of shifts nursing daily report completed (as per current hospital policy)	Pre-existing data collection of information from patient notes/RedCap Tool	Baseline, endline, sustainability assessment 6 months after the end of the project, each PDSA cycle
Completeness of nursing daily report form	% of completed compulsory fields on daily nursing report (i.e. patient ID, DOB/age, sex, name, patient assessment nursing report, nurse name, nurse signature, nurse designation)	Pre-existing data collection of information from patient notes/ RedCap Tool	Baseline, endline, sustainability assessment 6 months after the end of the project, each PDSA cycle
Completeness of other documentation	% change of each field on green registration card, yellow admission folder, Sierra Leone Early Warning Score (SLEWS)	Pre-existing data collection of information from patient notes/RedCap Tool	Baseline, endline, sustainability assessment 6 months after the end of the project, each PDSA cycle

#### Act

Based on the results from the analysis, changes or adjustments will be made to make improvements to the awareness drive (e.g. to WhatsApp text and posters). Findings of the awareness campaign will be disseminated through departmental meetings, QINDT, Hospital Newsletter, and WhatsApp.

### PDSA2: training package for nurses—improving knowledge, skills, and attitudes for quality clinical documentation

#### Aim

To introduce a training package for the nursing staff which will increase adherence to the hospital documentation standards.

#### Objective

Deliver, assess, and adjust a training package for improved nursing documentation.

#### Planned targets

Ninety percent of nursing staff of surgical department will have attended a training workshop.

Ninety percent of nursing staff will have had a minimum of two 1-to-1 support sessions.

#### Plan

The training package for nurses to improve the quality of nursing documentation in the surgical department will consist of two parts—a training workshop and ward-based 1-to-1 support sessions. The content, design and evaluation tools of this package will be co-developed by the research team, QINDT, and educational experts, using adult learning theories. To ensure the training package is culturally and contextually appropriate, and the workshop design and delivery methods used are effective, it will be piloted to a small group of staff who were not involved in the development process. The results of the pilot will be analysed, and the changes and adjustments to context and delivery of the training package will be made before finalising and approving the training package by the matron’s office.

#### Do

The training package will be delivered by the members of matron’s office and educational experts and coordinated by the research team. The entire process will be overseen by NB.

A 1-day workshop will be delivered to surgical nurses currently on the payroll including the matron’s team and nurses covering five general surgical wards and the trauma ward, as a mixture of didactive, interactive, and practical sessions. Each nurse will be required to have at least two 1-to-1 sessions that will be delivered on the ward by champions. During these sessions, three to four nursing daily notes from ward patients will be assessed on the application of the implemented nursing documentation framework for daily reporting, using a nursing documentation framework evaluation checklist and evaluation form (to be designed). Nurses will receive verbal and written clarification of any queries that arise during this session related to nursing documentation in general and nursing process framework in particular. There are 120 surgical nurses currently on the payroll. All will be invited to take part in the training workshop and 1-to-1 supportive supervision sessions.

#### Study

After 3 weeks from delivery of the training package, several outcomes to assess feasibility of delivering the intervention and capturing learning, process, and implementation will be collected and analysed as detailed in Tables 3 and 4.

#### Act

Based on the results from the analysis, any changes and adjustments will be made to the training package before being incorporated into regular new nursing staff induction sessions and continuing professional development programs. Findings of the training package assessment will be disseminated through presentations to the MoHS, surgical departmental meetings, QINDT, Hospital Newsletter, and WhatsApp.

### PDSA3: development of audit and feedback process as part of the QI process for improving and maintaining quality of nursing documentation (January–march 2021)

#### Aim

To co-develop and co-introduce an audit and feedback (A&F) process for nursing documentation by the research and clinical staff that is culturally and contextually appropriate and sustainable after the end of the intervention.

#### Objective

To co-introduce, assess, and adjust an audit and feedback (A&F) process, which reports results of quality of nursing documentation to the hospital QI committee and takes action to make improvements based on these results.

#### Plan

Development includes the identification and training of the staff by the matron’s office, development of the audit forms and a database to record the results, and determining frequency and sampling strategies of audit and feedback. A simple user-friendly ward-based audit form will be designed by QINDT that will be used for regular nursing documentation assessments. The A&F process will be structured around existing hospital/surgical department routine and changes that have been introduced during this project.

All staff assigned to participate in the A&F (i.e. champions) will be provided with a one half-day training session, to ensure they understand how to use the new tool and the whole audit process.

#### Do

The A&F process will be undertaken within PDSA1 and PDSA2 cycles as part of the ‘study’ component and led by the research team in collaboration with the QINDT. During this time, the process will be evaluated for appropriateness for long-term use and any changes made as necessary. After the end of PDSA1 and PDSA2 cycles, the A&F process will continue to be undertaken but now led by the matron team independently from the research team, according to the developed and agreed plan.

#### Study

After 2–3 audit and feedback cycles undertaken and led by the matron team (independently of the research team), several outcomes to assess feasibility of delivering the intervention and capturing process and implementation will be collected and analysed as detailed in Tables 3 and 4.

#### Act

Based on the results from the analysis, any changes or adjustments will be made if needed. Findings will be disseminated through departmental meetings, Hospital QI Committee meetings, Hospital Newsletter and WhatsApp.

## Evaluation

The evaluation phase will include three types of evaluation. First, we will assess the feasibility of delivering the intervention and capturing the outcomes needed to evaluate the implementation and effectiveness of this intervention. If feasible, second, we will evaluate the implementation and the impact of implementation strategies on the adoption, integration, and uptake of the intervention using implementation and process outcomes related to training. Third, we will evaluate the intervention’s effectiveness to improve quality of nursing documentation in this limited setting. All outcomes that will be collected as part of the pilot are described below.

### Outcomes

#### Feasibility outcomes

For each PDSA, we will assess the feasibility of delivery of the intervention and of assessing the outcome. As an implementation science project, PDSA cycles enable adjustment of the intervention if it is not delivered as intended. Each PDSA can stand alone; hence, progress to the next one is not contingent on delivery and assessment of outcomes being feasible in the preceding one. Issues in data capture will inform assessment methods for wider roll out of the project in multiple hospital settings. We will also assess feasibility of capturing data to evaluate the effectiveness of the project. If these data are feasible to collect, we will continue to collect to sample size to inform whether the intervention is effective and therefore should be tested in multiple hospital settings, in different contexts, and for different specialties.

#### Implementation and process outcomes

**Fig. 3:**
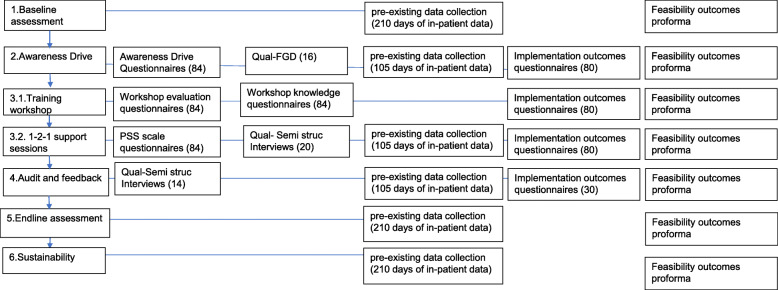
Evaluation of the QI project flow diagram. NB: implementation outcomes—acceptability, appropriateness, feasibility, Method of assessment: questionnaires—predefined, tested, and adapted instruments, Qual—qualitative interviews, FGD: focus group discussion, pre-existing data—collection of data from patients’ clinical records, ()-sample size feasibility outcomes for delivery of the intervention listed in Table 3

All outcomes that will be collected to evaluate the impact of implementation and implementation strategies are detailed in Table 4 and Fig. 3.

#### Effectiveness outcomes

The effectiveness outcome is a composite measure of the number of nursing daily report forms that was compliant and completed. Compliance is defined as the % of times a nursing daily report had been completed as required by the current hospital policy, and completeness is defined as % of nursing daily reports that have all compulsory fields completed (i.e. patient ID, DOB/age, sex, name, patient assessment nursing report, nurse name, nurse signature, nurse designation). All process outcomes that are required to calculate effectiveness outcomes are listed in Table 4 and Fig. 3.

#### Data collection

All data will be collected using RedCap software [48].

#### Sample size

Details of proposed sample sizes for all the outcomes are given below and captured in Fig. 3.

##### Awareness drive

A purposive sample of 80 doctors and nurses, as they enter the hospital, will be asked to take part in a survey capturing responses to the awareness drive WhatsApp messages and posters that are displayed. There are approximately 450 doctors and nurses present at the hospital during daytime shift. A sample of 80 will represent just under 20% of the total number of doctors and nurses, which is within a recommended sample size for pilots and feasibility assessments.

Up to a maximum of 16 health care staff will participate in two focus group discussions (FGDs) to assess their reactions to the awareness drive exercise, an equal number of nurses and doctors will form the two focus groups.

##### Training package

There are 120 nurses on the payroll in the surgical department. All nurses will be invited to participate. If we assume a 70% participation rate, we expect 84 nurses to take part. Approximately 20 nurses will be interviewed using qualitative semi-structured interviews after completing the training package. We will use quota sampling to ensure that we have surgical ward representation across all six wards. We will continue interviewing until thematic saturation has been reached, which we anticipate will be achieved after interviewing 20 nurses.

##### Audit and feedback

There will be approximately 30 hospital staff involved in the audit and feedback co-delivery phase. All of them will be invited to complete the implementation outcome questionnaires. Ten staff members will be interviewed using semi-structured interviews. We will use quota samplings to capture information from health care staff from the hospital at all levels and will include the following members: matron, hospital manager, working group members, two nurses in-charge, and five ward nurses.

For the *effectiveness outcome,* 10 surgical patient files from each of 6 wards will be selected at various time points (at baseline, end of each PDSA cycle, endline, and at the 6 month sustainability assessment time point) giving 60 files in total. Assuming that the average in-patient time of a surgical patient at Connaught Hospital is 7 days, it will provide 420 days of nursing notes for analysis at each time point. Assuming nurses’ daily notes are recorded on 50% of the days at baseline, the sample size of 210 provides 85% power to detect as significant (at the 5% level), an increase of 15% from baseline, in completion and compliance rate of nurses daily notes, including a set of four compulsory fields (Patient ID, name, sex, DOB/age) and application of a Nursing Documentation Framework (criteria to be defined).

### Analysis

The feasibility outcomes will be used to understand what changes needs to be made to the protocol in order to successfully deliver this study and replicate in other hospitals. The data on implementation (appropriateness, acceptability, and feasibility) of the interventions and implementation strategies piloted in this study will be captured and used to make changes to each component of the intervention (awareness drive, training package, and audit and feedback) that are needed for successful implementation. Any modifications that are required during this pilot will be monitored and described. The results will be discussed with the stakeholders who will be required to help with the interpretation of findings in the context and agree to any proposed changes [30].

For the effectiveness outcome, the change from baseline in the percentage of compliance and completeness rates will be calculated and formally tested using non-parametric (e.g. chi-square, Fisher’s exact, Mann-Whitney) and parametric (e.g. *t* test) statistical testing will be used as appropriate [49].

For all other outcomes, quantitative data collected through questionnaires (Fig. 3) will be presented descriptively. Qualitative data collected during interviews and FGDs (Fig. 3) will be recorded, transcribed, translated, and then coded and analysed using thematic analysis to capture the main themes. Thematic analysis will be used as it is a method of analysing qualitative data that seeks to identify and make sense of any themes found in the data [50].

## Discussion

This intervention will use evidenced-based QI methods and implementation frameworks to introduce and support improvement in nursing clinical documentation practice in a resource-poor setting in SSA. We will test for feasibility of developing, deploying, and testing this pilot intervention in one setting in Sierra Leone.

A key strength of this multifaceted QI project is that it has been co-designed from its inception with involvement of stakeholders at all levels within the health system. The project emphasis is on addressing specific needs of the local health system and design has been guided by a theory of change process. In addition, the formal use of implementation and improvement science methodologies will promote the collection of both process and implementation outcomes. It will allow exploration of the role of the implementation context and identify the role of implementation strategies within this context.

The results will generate knowledge to inform good nursing documentation practices for surgical patients in Sierra Leone and add to the body of evidence about the development and implementation of effective health system strengthening QI hybrid interventions in LMICs settings.

The project may not be applicable to every low-resource setting surgical context due to differences in healthcare systems. However, the application of implementation science concepts may facilitate transferability and adaptation to other settings. The project will generate findings on both the effectiveness of the intervention package for improving documentation, and on the relevance and applicability of implementation and improvement science concepts and methods within low resourced settings of perioperative care.

## Supplementary Information

**Additional file 1:.** Appendix 1: Details of composition and remit of the Quality Improvement for Nursing Documentation (QIND) Team.

## Data Availability

The datasets used and/or analysed during the current project will be available from the corresponding author on reasonable request.

## Abbreviations

QI: Quality improvement
LMIC: Low- and middle-income countries
HCS: Health Care Staff
ToC: Theory of Change
QIND: Quality Improvement of Nursing Documentation
QINDT: Quality Improvement of Nursing Documentation Team
PDSA: Plan Do Study Act
ERIC: Expert Recommendations for Implementation Change taxonomy of implementation
SPIRIT: Standard Protocol Items: Recommendations for Interventional Trial
SQUIRE: Standards for Quality Improvement Reporting Excellence
MoHS: Ministry of Health and Sanitation of Sierra Leone
SLEWS: Sierra Leone Early Warning Score
A&F: An audit and feedback
ID: Personal Identifier
BP: Blood pressure
AVPU: Alert-verbal-pain-unresponsive level of conscious
DOB: Date of birth
FGD: Focus group discussion
PSS: Perception to Supportive Supervision
