# Supplementary material for: Improving nursing documentation for surgical patients in a referral hospital in Freetown, Sierra Leone: protocol for assessing feasibility of a pilot multifaceted quality improvement hybrid type project
Source: Pilot Feasibility Stud. 2021 Jan 27;7:33. doi: 10.1186/s40814-021-00768-5 (PMC7839195; doi:10.1186/s40814-021-00768-5)
Supplement: Supplementary file 1 — Additional file 1:. Appendix 1: Details of composition and remit of the Quality Improvement for Nursing Documentation (QIND) Team. [file 40814_2021_768_MOESM1_ESM.docx]

Appendix 1:

Details of composition and remit of the Quality Improvement for Nursing Documentation (QIND) Team.

Quality Improvement for Nursing Documentation Team (QINDT) consisting of a working group and nursing champions. It is built upon an existing hospital quality improvement structure that was led by the surgical matron, now bringing ward nurses (champions) into the quality improvement process. The inclusion of the nurses into the QIND team will strengthen the link between management of the hospital and ward staff to promote greater planning and accountability of the quality improvement process.

The working group includes matrons, nursing officers, ward in-charges, representatives from the hospital management, partner organisations, senior and junior doctors, and research team members. It has been meeting on a monthly basis and will continue to do so for the duration of the project. It is expected that it will continue meeting after the intervention. All decisions taken by the group will be endorsed by the hospital management (Hospital Care Manger and the Hospital Senior Matron Team). The remit of the working group will be to:

- define and agree strategies to deliver the interventional bundle;
- review and contribute to the development of all materials needed for the intervention including WhatsApp messages, posters, training day resources, audit and feedback process;
- review current SOP related to nursing documentation and a Nursing Documentation Framework to be introduced as part of this intervention;
- support the delivery of training on nurse documentation and the audit and feedback process;
- review findings of documentation audits and monitor improvements in documentation completeness and compliance;
-
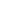
support dissemination of study findings to hospital leadership and Health Care Staff (HCS).

Ten nurse champions were selected on the advice and approval from the Hospital Senior Matron team from each clinical area (triage, Speciality Out-Patient Clinic, A&E, ICU, 5 surgical wards, trauma ward). Under the leadership of the Hospital Senior Matron team, and support from the working group, they are seen as the main advocates for change and their role is to represent the staff on the wards by working closely with the working group and research team. They are the key staff driving implementation and recommendations of the working group. Their duties include: to deliver peer support for the nurses on the ward; to lead 1-2-1 sessions; to lead ward audits and provide the working group with audit results in timely manner; to advocate for, and promote, good documentation practice by personal example and through continuous group and individual feedback as necessary.
